# Neurocognitive functioning and health-related quality of life in adult medulloblastoma patients: long-term outcomes of the NOA-07 study

**DOI:** 10.1007/s11060-020-03502-y

**Published:** 2020-05-04

**Authors:** Linda Dirven, Ralf Luerding, Dagmar Beier, Elisabeth Bumes, Christiane Reinert, Clemens Seidel, Matteo Mario Bonsanto, Michael Bremer, Stefan Rieken, Stephanie E. Combs, Ulrich Herrlinger, Corinna Seliger, Holger Kuntze, Regine Mayer-Steinacker, Annette Dieing, Claudius Bartels, Oliver Schnell, Astrid Weyerbrock, Sabine Seidel, Oliver Grauer, Minou Nadji-Ohl, Frank Paulsen, Michael Weller, Wolfgang Wick, Peter Hau

**Affiliations:** 1 Department of Neurology, Leiden University Medical Center, Leiden, The Netherlands; 2 Department of Neurology, Haaglanden Medical Center, The Hague, The Netherlands; 3 Wilhelm Sander-NeuroOncology Unit and Department of Neurology, University of Regensburg, Regensburg, Germany; 4 Department of Neurology, University Hospital Odense and Clinical Institute, University of Southern Denmark, Odense, Denmark; 5 Department of Oncology, Krankenhaus der Barmherzigen Brüder Regensburg, Regensburg, Germany; 6 Department of Radiation Oncology, University Hospital Leipzig, Leipzig, Germany; 7 Department of Neurosurgery, University Hospital, Lübeck, Germany; 8 Department of Radiation Oncology, Medical School Hannover, Hannover, Germany; 9 Department of Radiotherapy and Radiation Oncology, University Hospital Göttingen, Göttingen, Germany; 10 Department of Radiation Oncology, Technical University of Munich, Munich, Germany; 11 Institute of Radiation Medicine, Helmholtz Zentrum Münche, Oberschleißheim, Germany; 12 Deutsches Konsortium für Translationale Krebsforschung (DKTK), Partner Site Munich, Munich, Germany; 13 Division of Neurooncology, University of Bonn Medical Center, Bonn, Germany; 14 Department of Neurology, University Hospital Heidelberg, Heidelberg, Germany; 15 Neurooncology Program at the National Center for Tumor Diseases, German Cancer Research Center (DKFZ)/DKTK, Heidelberg, Germany; 16 Department of Neurology, University Hospital Mainz, Mainz, Germany; 17 Department of Medical Oncology, University Hospital Ulm, Ulm, Germany; 18 Department of Internal Medicine, Hematology and Oncology, Vivantes Klinikum am Friedrichshain, Berlin, Germany; 19 Department of Neurosurgery, University Hospital Magdeburg, Magdeburg, Germany; 20 Department of Neurosurgery, University Hospital Freiburg, Freiburg, Germany; 21 Department of Neurology, Knappschaftskrankenhaus, University of Bochum, Bochum, Germany; 22 Department of Neurology with Institute of Translational Neurology, University Hospital Münster, Münster, Germany; 23 Department of Radiation Oncology, Klinikum Stuttgart, Stuttgart, Germany; 24 Department of Radiation Oncology, University Hospital Tübingen, Tübingen, Germany; 25 Department of Neurology, University Hospital and University of Zurich, Zurich, Switzerland

**Keywords:** Brain tumor, Medulloblastoma, Cognition, Quality of life, Patient-reported outcome, Toxicity

## Abstract

**Background:**

Combined radiochemotherapy followed by maintenance chemotherapy with cisplatin, lomustine and vincristine within the NOA-07 study resulted in considerable short-term toxicity in adult medulloblastoma patients. Here we investigated the long-term impact of this treatment, focusing on neurocognitive functioning and health-related quality of life (HRQoL).

**Methods:**

Neurocognitive functioning and HRQoL scores over time were determined, and differences between the post-treatment and follow-up assessments were calculated up to 18 months for neurocognition and 60 months for HRQoL.

**Results:**

28/30 patients were analyzed. The three preselected HRQoL scales (role, social and cognitive functioning) showed improved scores, to a clinically relevant extent (≥ 10 points), compared to post-treatment levels up to 30 months, but decreased afterwards. Z-scores for verbal working memory were worse during follow-up compared to post-treatment scores and remained impaired during 18 months follow-up (i.e. z-score below − 1 standard deviation). Attention was impaired post-treatment, and remained impaired to a clinically relevant extent during follow-up. Coordination/processing speed and lexical verbal fluency improved compared to post-treatment scores, and remained within the normal range thereafter. Other tests of verbal fluency were stable over time, with z-scores within the normal range.

**Conclusions:**

This long-term follow-up study showed that the NOA-07 treatment regimen was not associated with a deterioration in HRQoL in the post-treatment period. Verbal working memory deteriorated, while other neurocognitive domains did not seem to be impacted negatively by the treatment.

**Electronic supplementary material:**

The online version of this article (10.1007/s11060-020-03502-y) contains supplementary material, which is available to authorized users.

## Introduction

Medulloblastoma is a rare entity in adults, with an incidence of 0.6 cases per million persons per year [1]. Within the adult population, the disease typically affects young adults, at a median age between 20 and 40 years [2–4]. Current standard of care consists of surgery, radiotherapy and/or chemotherapy, but newer therapies including immunotherapy and targeted therapy are being explored, particularly in the pediatric population [5]. Despite multimodal anti-tumor treatment, tumor recurrence is inevitable in part of the patients. Recently reported 5- and 10-year overall survival rates ranged between 74 and 82% [1, 4, 6] and between 65 and 67%, respectively [1, 6], and depend on clinical characteristics of patients and the histological and genetic profile of the tumor. [3, 4, 6–8].

Medulloblastoma patients may suffer from a variety of symptoms, depending on the tumor location [9, 10] and its subsequent treatment. Anti-tumor treatment may alleviate the symptoms caused by the tumor, but may also result in early and late toxicity [2, 3, 11]. In the short-term, patients with medulloblastoma may experience problems with coordination and gait. Increased intracranial pressure may cause symptoms such as headache, nausea and vomiting. When metastasized to the cerebrospinal fluid, back pain, muscle weakness, and loss of bladder and bowel function are frequent problems. Common acute toxicities in adult patients with medulloblastoma treated with radiochemotherapy are bone marrow suppression, polyneuropathy and ototoxicity [2].

As medulloblastoma patients have a relatively long survival, the impact of treatment on the patients’ long-term functioning and well-being should also be considered during treatment decision-making. Most of the long-term adverse effects are not self-limiting. One of the most relevant long-term complications of radiotherapy in brain tumor patients is neurocognitive dysfunction [12], which may subsequently interfere with a patients’ functioning in daily life, particularly role and social functioning. Although it has been shown that a large proportion of adult medulloblastoma patients have impaired neurocognitive functioning shortly after diagnosis, particularly in the domains learning, memory, and executive function [13], data on the short- and long-term impact of treatment on outcomes such as neurocognition and HRQoL are limited. One study reported impaired executive function, weakness, ataxia, depression and anxiety about nine years after radiotherapy [14]. A review on core deficits and quality of survival in childhood medulloblastoma survivors showed long-term neurological and sensory (e.g. hearing loss) problems, endocrine dysfunction, neurocognitive impairments (particularly in the domains information processing speed, attention and working memory) and psychosocial problems, particularly with role and social functioning [15]. In knowledge of these possible short- and long-term side effects, information on both the quantity and quality of survival should be available when informing patients on the benefits and risks of a treatment strategy. The NOA-07 study investigated a combined radiochemotherapy of the neuroaxis with a boost to the posterior fossa in combination with vincristine, followed by maintenance chemotherapy with cisplatin, lomustine and vincristine with the main endpoint of toxicity-related treatment terminations after four cycles of adjuvant chemotherapy, and the acute toxicity profile in adult patients. Previously, the short-term impact of treatment in this study was investigated, showing considerable toxicity during active treatment, but improvements in HRQoL and neurocognitive functioning [2]. Here we describe the long-term disease burden, up to five years after diagnosis, of adult medulloblastoma patients treated in the NOA-07 study in terms of neurocognitive functioning and health-related quality of life (HRQoL), which were secondary endpoints.

## Methods

### Study population

Adult (age ≥ 21 years) medulloblastoma patients with a Chang stage T1–4 and M0 or M1 were included. Further details on the study population are available elsewhere [2]. The study was approved by the ethical review boards of all participating centers, and patients provided written informed consent before participation.

### Study design and treatment

NOA-07 was a prospective study in which all patients received photon craniospinal irradiation (35.2 Gy in 22 fractions of 1.6 Gy, with a posterior fossa boost of 55 Gy in fractions of 1.8 Gy) in combination with vincristine (1.5 mg/m^2^ per week), followed by a maximum of eight six-week cycles of cisplatin (70 mg/m^2^; day 1), lomustine (75 mg/m^2^; day 1) and vincristine (1.5 mg/m^2^; days 1, 8 and 15). Further details on the treatment schedule have been published previously [2].

### Outcomes

Primary endpoints of NOA-07 study were toxicity-related treatment terminations after four cycles of adjuvant chemotherapy, and acute toxicity profile. Event-free, progression-free and overall survival were secondary endpoints, as were neurocognitive functioning and HRQoL.

#### Neurocognitive functioning

A neurocognitive test battery was used to assess several relevant domains. Since the representations of working memory, word fluency and attention involve cerebellar functions to a substantial level [16–19], these domains were chosen. The digit span forward and backward test from the German version of the Wechsler Adult Intelligence Scale-Revised [20] was used to assess attention span and verbal working memory. Trail Making Test Parts A and B [21] were used to measure coordination/processing speed and divided attention. Lexical verbal fluency was assessed with the Controlled Oral Word Association (COWA) test [22], while semantic verbal fluency was measured with the Regensburger Wortfluessigkeitstest [23]. To reduce practice effects, tests were chosen with minimal effects by repeated measurements [24]. Raw test scores were converted into z-scores, adjusting for age, sex and education, using population-based, normative data (means and standard deviations), provided by the respective test author in the test manual. A difference in z-score of -1 standard deviation (SD) was chosen as cut-off to indicate impaired performance of patients as compared to the norm population, as well as to indicate a clinically relevant change over time [2, 19, 25]. Neurocognitive functioning was evaluated between resection and the start of combined radiochemotherapy and after adjuvant chemotherapy, and subsequently every three months for up to 24 months, after which neurocognition was assessed every six months up to 60 months.

#### HRQoL

The EORTC core Quality of Life Questionnaire (QLQ-C30, version 3.0) was used in combination with the brain cancer module (QLQ-BN20). Both instruments have shown robust psychometric properties [26–28]. Following the EORTC scoring manual, raw item scores were aggregated and transformed into linear scales ranging from 0 to 100 [29]. For functional scales, a higher score represents better functioning, while for the symptom scales/items a higher score represents a higher level of symptoms. A difference ≥ 10 points on any scale/item was deemed clinically relevant [30]. HRQoL was evaluated between resection and the start of combined radiochemotherapy, after the conclusion of adjuvant chemotherapy, and subsequently every 3 months thereafter for up to 24 months, after which HRQoL was assessed every six months up to 60 months. Based on clinical experience, long-term social outcome was considered most relevant to adult medulloblastoma patients, who are typically younger adults with an active family and social life. Therefore, three scales were selected for primary analysis: role functioning (RF), cognitive functioning (CF) and social functioning (SF). All other scales/items were analyzed on an exploratory basis.

### Statistical analysis

#### Descriptive statistics

Sociodemographic and clinical characteristics of the patients included in this study were reported by means of descriptive statistics, as were (changes in) neurocognitive functioning and HRQoL scores. Patients with data available at the moment of disease progression were included in the analyses. Compliance with neurocognitive and HRQoL assessments as determined for each included follow-up moment was calculated by dividing the number of completed tests or HRQoL forms at a specific time point by the number of expected test scores or forms at that moment.

#### Longitudinal course of neurocognitive functioning and HRQoL

Graphs were constructed to assess neurocognitive functioning and HRQoL scores over time, and a visual description provided accordingly. Wilcoxon signed rank test was used to calculate whether the differences between the post-treatment and follow-up neurocognitive and HRQoL scores were statistically significant, but these analyses should be considered exploratory due to the limited number of patients. In addition, the percentage of patients with impaired neurocognitive functioning was calculated at each time point, separately for each domain.

A sub-analysis was performed in those patients with follow-up HRQoL data beyond 36 months, to gain more insight into their HRQoL trajectory. To do so, an average score during treatment was compared with the average score during long-term follow-up, which was defined as the average in a specific HRQoL scale score between 36 and 60 months follow-up.

All statistical analyses were done with SPSS version 25.0 (Armonk, NY: IBM Corp), and a p-value < 0.05 was considered statistically significant.

## Results

A total of 28 out of 30 eligible patients completed at least one valid HRQoL questionnaire or underwent neurocognitive testing and were included in this analysis. Included patients were on average 37 (SD 10) years of age, the majority (61%) was male, and the median KPS at inclusion was 90. Most tumors were in the classic and desmoplastic/nodular entities. All patients had undergone resection and received combined radiochemotherapy. Eighty-nine percent of patients received at least one cycle of chemotherapy (median 6; range 0–8). During follow-up, five patients had disease progression after a median of 1.8 years (range 1.1–4.5), and the 3- and 5-year event-free survival rate was 66.6% each. The progression-free and overall survival rates were 66.6% for PFS after 3- and 5-years, and 70% for OS after 3- and 5-years, respectively (Table 1).

**Table 1:** Baseline sociodemographic and clinical characteristics of medulloblastoma patients in the NOA-07 study who completed at least one valid health-related quality of life form or underwent neurocognitive testing at least once

	Baseline characteristics (n = 28)
Age in years at diagnosis	
Mean (SD; range)	37 (10; 22–54)
Gender, no. (%)	
Male	17 (61%)
Female	11 (39%)
KPS, median (range)	90 (50–100)
Histopathological entity, no. (%)	
Classic	12 (43%)
Desmoplastic/nodular	14 (50%)
Other	2 (7%)
Molecular entity, no. (%)	
SHH-driven, p53wt	18 (64.3%)
SHH-driven, p53mut	–
WNT-driven	4 (14.3%)
Group 3	–
Group 4	5 (17.9%)
Not available	1 (3.5%)
Radiochemotherapy completed	28 (100%)
Adjuvant chemotherapy received	25 (89%)
Chemotherapy cycles, median (range)	6 (0–8)
Disease progression, no. (%)	5 (18%)
Progression-free survival in years, median (range); (n = 5)	1.8 (1.1–4.5)
5-year overall survival rate (%)	70%

*SD* standard deviation, *KPS* Karnofsky Performance Status

### Compliance with HRQoL and neurocognitive assessments

**Fig. 1:**
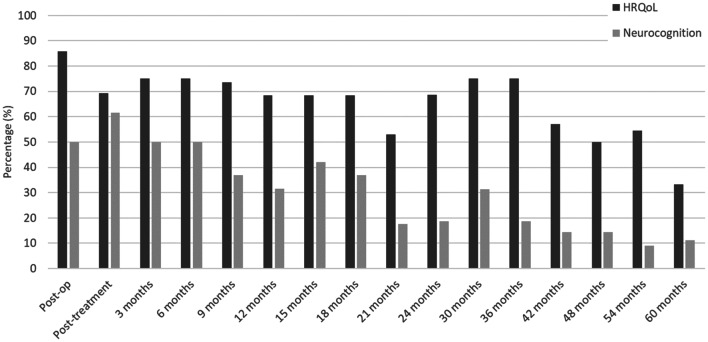
Compliance with health-related quality of life assessments and neurocognitive testing

At the pre-treatment assessment, compliance with HRQoL assessments was 86%, which decreased over time to 33% after 5 years of follow-up (Fig. 1). The average compliance rate for HRQoL over time was 65%. For neurocognitive testing these percentages were slightly lower: 50% compliance at the pre-treatment assessment, which dropped to 11% after 5 years of follow-up. Because compliance was < 30% from 18 months onwards for neurocognitive testing, these analyses were conducted up to 18 months only. Reasons for drop-out were the decision of the investigator to terminate treatment (8%), toxicity (29%), withdrawal of consent (29%), non-compliance (8%) and progressive disease (25%). See Supplemental Table 1 for the clinical characteristics of patients included in the 18-month neurocognitive assessments.

### Health-related quality of life

**Fig. 2:**
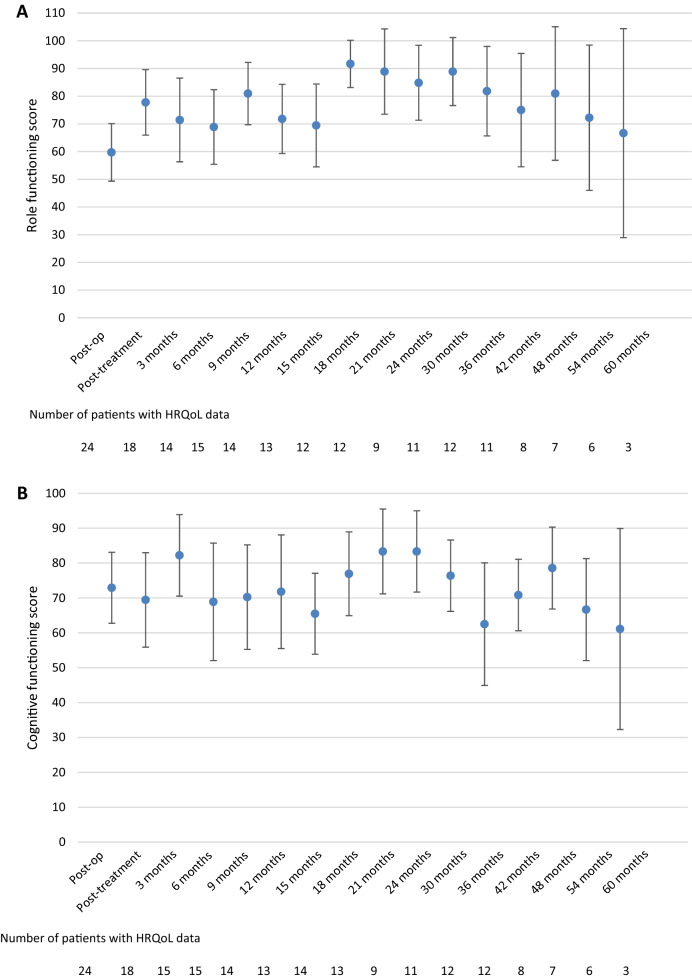

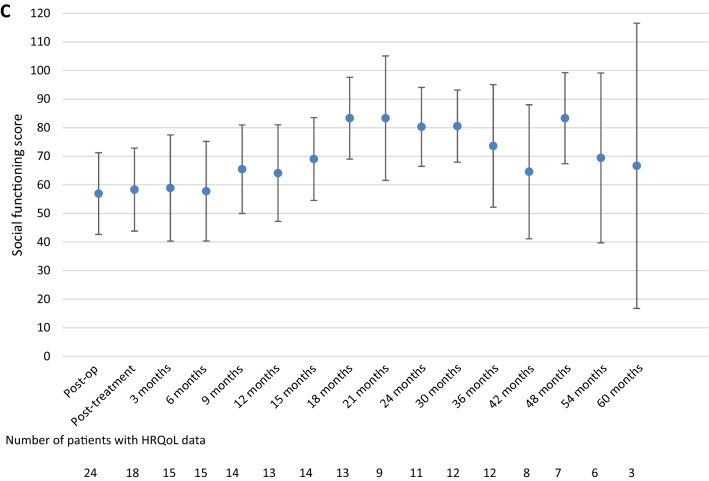
Mean health-related quality of life scores and its 95% confidence interval over time, from the postoperative assessment (before radiochemotherapy) up to five years of follow-up for the preselected scales **a** role functioning, **b** cognitive functioning and **c** social functioning

As previously reported [2], treatment resulted in an improvement of role, cognitive and social functioning on the group level [2]. Compared to post-treatment scores, none of the follow-up scores was significantly different for any of the preselected scales. Although the numbers of patients decreased over time, resulting in a limited number of patients available for analysis (< 10 patients beyond 36 months follow-up), the results show that role functioning did improve to a clinically relevant extent compared to post-treatment levels up to 30 months, but decreased afterwards. Similar trends were observed for cognitive and social functioning (Fig. 2), and also for the exploratory HRQoL scales/items (data not shown).

To gain more insight into the HRQoL trajectory of those patients with available long-term follow-up data (i.e. ≥ 36 months), we compared scores during treatment with those during long-term follow-up. For the 12 eligible patients, we found that the average score for social functioning was higher at long-term follow-up than during treatment (77 vs. 50, p = 0.006). For role and cognitive functioning, treatment and long-term follow-up scores were not different; respectively 69 vs. 81 (p = 0.126) for role functioning, and 73 vs. 69 (p = 0.397) for cognitive functioning.

### Neurocognitive functioning

The median number of tests on which patients had impaired neurocognitive functioning was 3 before treatment, 3.5 after treatment and ranged between 1.5 and 3.5 during follow-up (Table 2).

**Table 2:** Percentage of patients with impaired neurocognitive functioning (z-score < − 1 SD) over time, separately for each domain

	Pre-treatment	Post-treatment	3 m FU	6 m FU	9 m FU	12 m FU	15 m FU	18 m FU
Trail Making Test-A	8/14 (57%)	10/16 (63%)	5/10 (50%)	4/10 (40%)	1/7 (14%)	3/6 (50%)	2/7 (29%)	1/6 (17%)
Trail Making Test-B	9/14 (64%)	11/16 (69%)	7/10 (70%)	8/10 (80%)	4/7 (57%)	2/6 (33%)	4/7 (57%)	3/6 (50%)
Digit span forward	1/14 (7%)	3/16 (19%)	9/10 (90%)	6/10 (60%)	6/7 (86%)	3/6 (50%)	6/8 (75%)	5/7 (71%)
Digit span backward	2/14 (14%)	5/16 (31%)	8/10 (80%)	6/10 (60%)	3/7 (43%)	4/6 (67%)	4/8 (50%)	5/7 (71%)
Lexical fluency	10/13 (77%)	10/14 (71%)	2/10 (20%)	3/10 (30%)	2/7 (29%)	1/6 (17%)	3/8 (38%)	3/7 (43%)
Animal naming	5/14 (36%)	8/16 (50%)	1/10 (10%)	4/10 (40%)	1/7 (14%)	1/6 (17%)	4/8 (50%)	3/7 (43%)
Food naming	6/13 (46%)	7/14 (50%)	2/10 (20%)	4/10 (40%)	2/7 (29%)	1/6 (17%)	3/8 (38%)	2/7 (29%)
Median (range) tests impaired	3 (1–6)	3.5 (0–7)	3.5 (1–5)	3.5 (1–6)	3 (0–5)	1.5 (0–5)	3.5 (0–6)	3 (0–6)

#### Attention span and verbal working memory

**Fig. 3:**
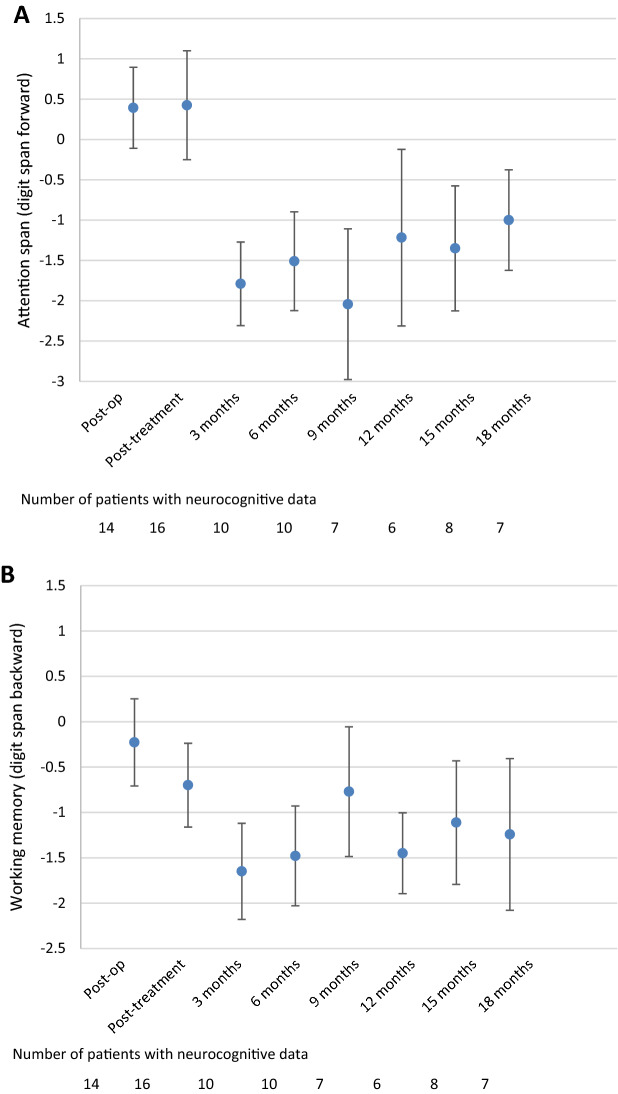

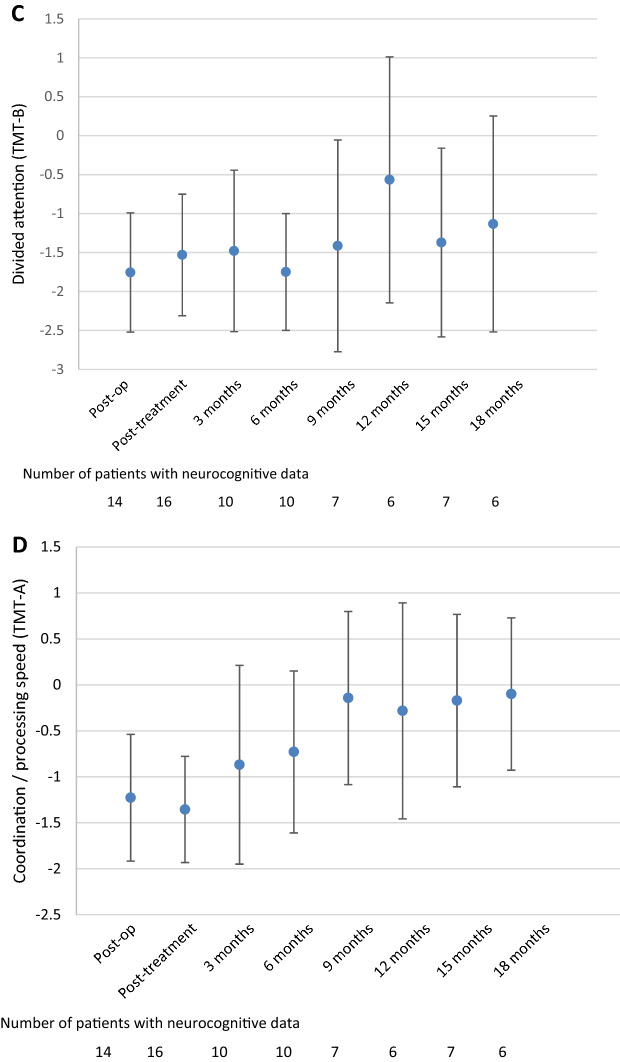

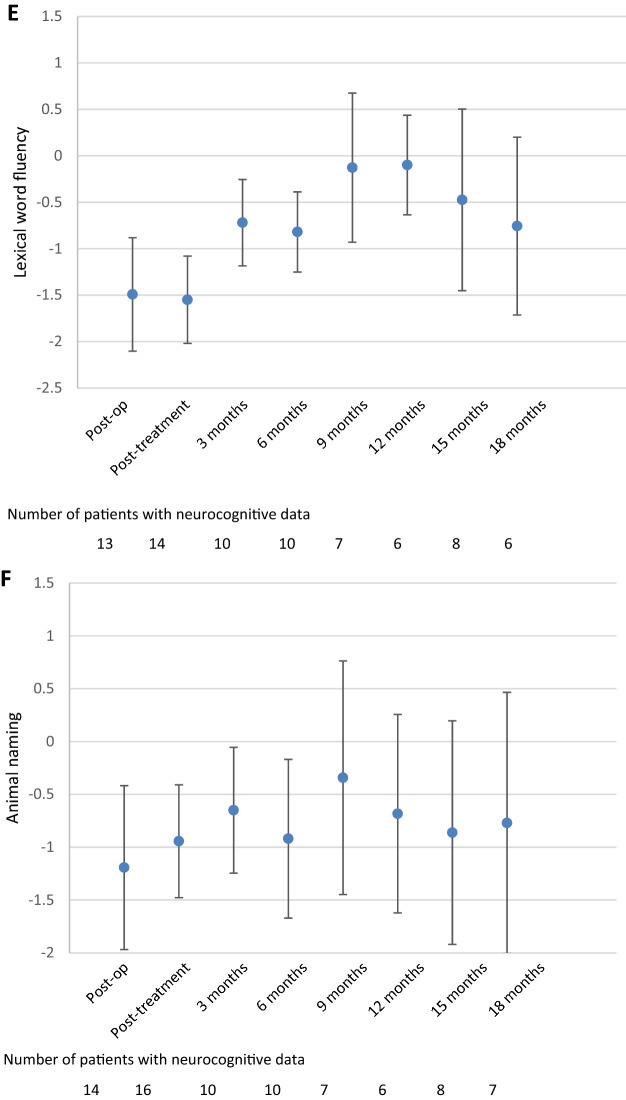

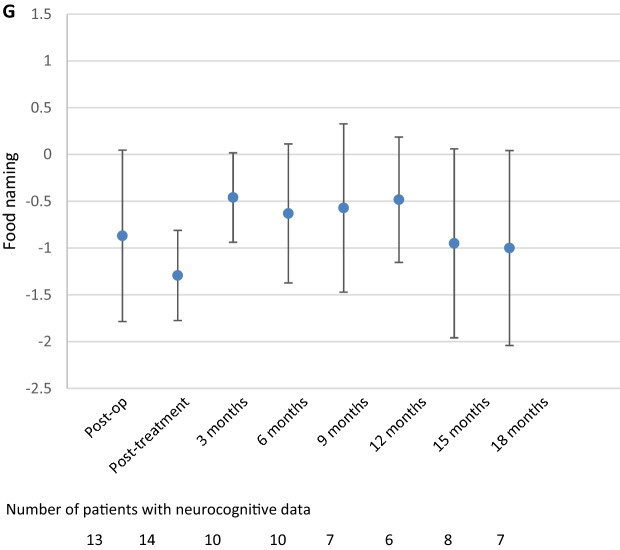
Mean Z-scores and 95% confidence interval for the different neurocognitive tests over time; **a** Attention span (digit span forward), **b** working memory (digit span backward), **c** divided attention (Trail Making Test B), **d** coordination/processing speed (Trail Making Test A), **e** lexical word fluency, **f** animal naming, and **g** food naming

The digit span forward test showed that the mean z-scores during follow-up were lower than the post-treatment z-scores: 0.4 during the post-treatment assessment versus − 1.79 at 3 months follow-up (p = 0.012) and − 1 at the 18-month assessment (p = 0.027), indicating the bottom of normal performance. The amelioration between the 3- and 18-month assessment was significant (p = 0.043), but not clinically relevant (Fig. 3a). The findings on group level are supported by the findings on individual patient levels, showing that the percentage of impaired patients increased from 19% post-treatment to between 50 and 90% during follow-up (Table 2).

Whereas the digit span backward test was in the normal range before and directly after the treatment, working memory was impaired (z-score > − 1) at all time points except after 9 months follow-up (z-score = − 0.8) (Fig. 3b). Differences were statistically significant and clinically relevant between the post-treatment z-score and the 3- and 6-month follow-up assessment z-scores (p = 0.027 and p = 0.028, respectively). On the individual patient level, the results also indicated that the majority of patients had impaired working memory over time, except after 9 months of follow-up (43%; Table 2).

#### Divided attention and coordination/processing speed

Performance in divided attention (TMT B) did not change during follow-up when compared to the post-treatment assessment (Fig. 3c). Although the z-score was close to normal at the 18 months follow-up assessment (z-score = − 1.1), attention remained impaired during follow-up. On the individual level it was also shown that the majority of patients, except for the 12-month assessment (33% impaired), had impaired divided attention.

Coordination/processing speed (TMT A) was impaired during the post-treatment assessment (z-score = − 1.4), but improved into the normal range at 3 months follow-up (z-score = − 0.9, p = 0.028) and remained stable afterwards (Fig. 3d). Indeed, during the post-treatment assessment, 63% of patients had impaired coordination/processing speed, which decreased to 17–50% of patients during follow-up.

#### Verbal fluency

Lexical word fluency was impaired to a clinically relevant extent before and directly after treatment, but improved during follow-up, with scores ranging between − 0.1 and − 0.8 (Fig. 3e), which are in the normal range. Compared to the post-treatment assessment, patients had significantly and clinically relevant better z-scores at 9-, 12- and 15-months follow-up (all p < 0.05). This was also reflected on the individual patient level, where 71% of patients had impaired lexical word fluency during the post-treatment assessment, and between 17 and 43% during follow-up (Table 2).

For the animal naming test, the post-treatment score was in the normal range, which was continued during follow-up, with no significant differences between the post-treatment and follow-up z-scores (Fig. 3f). Although in the normal range, results on the individual level showed that half of patients had impaired animal naming during the post-treatment assessment, and this percentage ranged between 10 and 50% during follow-up (Table 2).

Although the post-treatment score for food naming was impaired on the group level, follow-up scores were all in the normal range, ranging between − 0.5 (3 months) and − 1 (18 months) (Fig. 3g), although this improvement was neither statistically significant nor clinically relevant. Similarly, on the individual level it was shown that 50% of patients had impaired food naming during the post-treatment assessment, and this percentage decreased (ranging between 17 and 40%) during follow-up (Table 2).

## Discussion

Treatment of adult medulloblastoma patients in the NOA-07 study with combined radiochemotherapy of the neuroaxis followed by maintenance chemotherapy with cisplatin, lomustine and vincristine has been shown, in the short-term, to result in considerable toxicity during active treatment, however paralleled by improvements in HRQoL and neurocognitive functioning [2]. Here, we present mid- to long-term analyses on HRQoL and neurocognitive functioning, with the goal to better understand the impact of radiochemotherapy on the patients’ longer term functioning and well-being. In general, this analysis showed that the NOA-07 treatment regimen on group level did not result in a (further) deterioration of HRQoL or neurocognitive functioning, except for verbal working memory, in the post-treatment period. It should be noted though, that a substantial part of the study population did experience impaired neurocognitive functioning during follow-up as shown by the analysis on the individual patient level (Table 2).

The impact of treatment on neurocognition and HRQoL in adult medulloblastoma patients is understudied. A recent study reported impaired neurocognitive functioning (learning and memory, and executive function) shortly after diagnosis [13], while another study reported that adult medulloblastoma patients had problems with executive dysfunction, weakness, ataxia, depression or anxiety about nine years after radiotherapy [14]. These long-term sequelae may subsequently have a negative impact on HRQoL. In NOA-07 study, HRQoL scores remained lower than in healthy controls [2], as did neurocognitive scores. In pediatric patients, severe and persistent deficits in neurocognitive functioning were observed after median 10 years posttreatment with radiotherapy and/or chemotherapy, with significant impairments in aspects of daily living [31, 32], but not HRQoL [32]. Another study reported no long-term impact on HRQoL 6 years after proton therapy, although scores were below those of healthy controls [33]. Besides neurocognitive impairments, survivors of childhood medulloblastoma also often experience neurological deficits such as hearing loss and endocrine dysfunction [15], which have shown to negatively impact HRQoL in patients with pituitary adenoma [34] or vestibular schwannoma [35].

As medulloblastoma in adults typically affects younger patients, long-term social outcome of treatment is of main interest. This study showed that social functioning as measured with the EORTC QLQ-C30, reflecting the impact of disease and treatment on the patient’s family and social life, improved over time, although scores remained well below that of the general population [36]. Other important survivorship issues [37], such as career opportunities and limiting of life plans/goals are currently investigated only in childhood medulloblastoma [38]. Certainly, a broader approach would provide more insight into the long-term disease burden in this disease. Adding items from the EORTC item Library [39] to the core HRQoL questionnaire and brain tumor module would be an option to fulfil this goal.

In the 18-month post-treatment period of NOA-07, we observed clinically relevant dysfunction in the domains divided attention and working memory, which remained stable over time. Indeed, over time, the majority of patients experienced impairments in these domains. Previous studies have shown that isolated cerebellar lesions can cause impairments in working memory [19]. There is also mounting evidence that the cerebellum not only participates in working memory, but also in other higher-order cognitive tasks such as executive processing, verbal fluency and planning as well as linguistic and affective performance [16–18]. An anatomical substrate for these functions is a cerebellar feedback loop through the thalamus and prefrontal and inferior parietal lobule of the parietal cortex as reported for primates. Therefore, a major cause of neurocognitive dysfunction in these tumors seems tumor location. Nevertheless, in children it has been shown that craniospinal radiotherapy with posterior fossa boost has an additional severe negative impact on neurocognition [40–43]. This effect may be less pronounced in young adults or too early to be detected. In patients with low-grade glioma, the presence of a tumor had the most detrimental impact on the patient’s neurocognitive functioning. Treatment with radiotherapy in fractions > 2 Gy did result in neurocognitive dysfunction after a median of six years after diagnosis [44], and after a median of 12 years after diagnosis also fractions ≤ 2 Gy were associated with neurocognitive dysfunction [12]. Radiotherapy dose reduction has impact on reducing neurocognitive toxicity. Long-term radiotherapy-induced structural sequelae as leukoencephalopathy and radiation-induced vasculopathy relate to neurocognitive deficits [45, 46]. Neurocognitive impairment after cranial irradiation is more severe in children, but also prevalent in adults [14]. The boost radiotherapy volume can be reduced to the tumor bed only in children without losing efficacy in comparison to posterior fossa irradiation, if a dose of 50 Gy is sustained [47, 48]. Also, a reduction of the craniospinal dose to 23.4 Gy in combination with chemotherapy has been investigated in pediatric trials and showed non-inferior efficacy [49]. However, both approaches were not investigated in adults in a prospective manner so far. As there is mounting evidence from pediatric trials that radiotherapy dose is associated with neurocognitive dysfunction, efforts to reduce radiotherapy dose and boost volume should be undertaken in well-selected adult medulloblastoma patients, without compromising tumor control.

The association between neurocognitive functioning and HRQoL has already been shown in patients with brain tumors, but not in adults with medulloblastoma, with worse HRQoL scores in patients with impaired neurocognitive functioning [50]. Although the limited sample size in NOA-07 refrained us from analyzing whether a change in neurocognitive functioning was associated with a change in HRQoL, we would expect that impaired neurocognition has a negative impact on the patients’ HRQoL, particularly on the longer term.

Due to the small sample size and decreasing compliance over time, a common problem with neurocognitive and HRQoL assessments during follow-up in prospective brain tumor studies [51–54], long-term analysis of neurocognitive functioning beyond 18 months was not possible. In general, longer term results for both neurocognition and HRQoL should be interpreted with caution, as patients with better prognosis or good response to treatment are typically overrepresented during follow-up [55–57]. The finding that HRQoL improved up to 30 months after which a deterioration was observed, is likely caused by drop-out of patients over time, and is not necessarily a reflection of the development of long-term toxicity. This is supported by our sub-analysis in those patients with follow-up ≥ 36 months, showing that social, role and cognitive functioning improved or remained stable in the post-treatment period. Another limitation is that this study reflects a trial population, with stringent inclusion criteria, hampering generalizability of the results to the entire adult medulloblastoma patient population. Moreover, both neurocognition and HRQoL are impacted by other factors than treatment alone, including age, tumor course, comorbidity and supportive treatment [58, 59]. According to this, in glioma patients, progression was found to be the main cause of deterioration in HRQoL, and not treatment [60]. Due to the limited number of patients with progression and available neurocognitive or HRQoL data at the moment of progression, the impact of progression of the patients’ functioning and well-being could not be evaluated in this study. Similarly, subgroup analyses were not considered meaningful because of the small sample size, while certain aspects may be associated with neurocognitive functioning and possibly HRQoL. For example, different molecular subtypes and germline polymorphisms [61] have shown to be associated with neurocognitive functioning in childhood medulloblastoma patients, and patients who did or did not complete the entire treatment regimen may also differ in their level of functioning. Lastly, changes in neurocognition and HRQoL on the individual patient level were not considered meaningful due to the limited number of patients available for analysis, even though these analyses may provide more detailed information on the impact of treatment. Indeed, presenting data on group level only may result in an underestimation of the actual problems.

In conclusion, posttreatment data of the NOA-07 study showed that combined radiochemotherapy followed by maintenance chemotherapy in general does not result in a deterioration of the functioning and well-being of adult medulloblastoma patients on the medium long term. Nevertheless, a larger adequately powered trial with a higher number of patients is needed to confirm these findings, and to prove the benefits of reduced radiotherapy dose and boost volume in adult medulloblastoma patients. Currently, a large international trial, EORTC 1634-BTG, is being developed at the EORTC to investigate a personalized intensity-modulated therapy in post-pubertal patients with newly-diagnosed medulloblastoma, in which neurocognitive functioning and HRQoL are included as major secondary outcomes.

## Electronic supplementary material

Below is the link to the electronic supplementary material.

Supplementary file 1 (DOCX 13 kb)
