# Supplementary material for: Neurocognitive functioning and health-related quality of life in adult medulloblastoma patients: long-term outcomes of the NOA-07 study
Source: J Neurooncol. 2020 May 4;148(1):117–30. doi: 10.1007/s11060-020-03502-y (PMC7280359; doi:10.1007/s11060-020-03502-y)
Supplement: Supplementary file 1 — (DOCX 13 kb) [file 11060_2020_3502_MOESM1_ESM.docx]

**Supplemental Table 1. Baseline sociodemographic and clinical characteristics of medulloblastoma patients in the NOA-07 study who underwent neurocognitive testing at the 18-month assessment**

|  | **Baseline characteristics**  **(n=7)** |
| --- | --- |
| Age in years at diagnosis  Mean (SD; range) | 37 (6; 27-43) |
| Gender, no. (%)  Male  Female | 4 (57%)  3 (43%) |
| KPS, median (range) | 90 (70-100) |
| Histopathological entity, no. (%)  Classic  Desmoplastic/nodular  Other | 3 (43%)  3 (43%)  1 (14%) |
| Molecular entity, no. (%)  SHH-driven, p53wt  SHH-driven, p53mut  WNT-driven  Group 3  Group 4  Not available  Radiochemotherapy completed | 5 (71.4%)  0  0  0  1 (14.3%)  1 (14.3%)  7 (100%) |
| Adjuvant chemotherapy received | 7 (100%) |
| Chemotherapy cycles, median (range) | 8 (6-8) |
| Disease progression, no. (%) | 2 (29%) |
| Progression-free survival in years, median (range); (n=5) | 3.2 (1.8-4.5) |
| 5-year overall survival rate (%) | 5 (71.4%) |

*SD, standard deviation; KPS, Karnofsky Performance Status*
